# New artery of knowledge: 3D models of angiogenesis

**DOI:** 10.1530/VB-19-0026

**Published:** 2019-12-03

**Authors:** Eleonora Zucchelli, Qasim A Majid, Gabor Foldes

**Affiliations:** 1 National Heart and Lung Institute, Imperial College London, London, UK; 2 Heart and Vascular Center, Semmelweis University, Budapest, Hungary

**Keywords:** angiogenesis, endothelial cells, 3D assays

## Abstract

Angiogenesis and vasculogenesis are complex processes by which new blood vessels are formed and expanded. They play a pivotal role not only in physiological development and growth and tissue and organ repair, but also in a range of pathological conditions, from tumour formation to chronic inflammation and atherosclerosis. Understanding the multistep cell-differentiation programmes and identifying the key molecular players of physiological angiogenesis/vasculogenesis are critical to tackle pathological mechanisms. While many questions are yet to be answered, increasingly sophisticated *in vitro*, *in vivo* and *ex vivo* models of angiogenesis/vasculogenesis, together with cutting-edge imaging techniques, allowed for recent major advances in the field. This review aims to summarise the three-dimensional models available to study vascular network formation and to discuss advantages and limitations of the current systems.

## Introduction

The major role of the vascular system is to supply sufficient levels of oxygen and nutrients to the bodily organs. Naturally, any disruption to this system manifests itself as a host of diseases including, but not limited to, stroke, peripheral artery disease and other ischaemic cardiovascular diseases. Furthermore, the unregulated expansion of the vasculature during tumour angiogenesis can have detrimental consequences resulting in cancer metastasis. However, repair of the vasculature can hold immense therapeutic potential in tissue engineering approaches. Therefore, a greater understanding of the underlying endothelial biology that governs vessel formation and remodelling is required; this is a complex process driven by a combination of cells in tandem with an array of factors and signalling cascades. Moreover, other than blood vessels, another type of vasculature exists, which form the lymphatic network. Lymphatic vessels are unidirectional, blind-ended capillaries, which arise from the venous vasculature, and they are also formed by endothelial cells. The lymphatic system has the fundamental role of draining interstitial fluids from the tissues, and it is also involved in the immune surveillance of the body (1, 2). A great variety of assays has been developed to study blood and lymphatic vessel formation, each with advantages and limitations. These bioassays allowed to study the biology of angiogenesis and vasculogenesis, to test drugs that can modulate angiogenesis in normal and pathological conditions, and to discover mechanisms of lymphangiogenesis (3). This review aims to focus on 3D models of blood vessel formation.

## Angiogenesis and vasculogenesis

Vasculogenesis refers to the *de novo* formation of the primitive vascular plexus and the heart during embryonic development, via the differentiation of endothelial cell precursors (hemangioblasts) into endothelial cells (4). Angiogenesis refers to the formation of new vessels from pre-existing ones, thus remodelling of the primitive vascular plexus during and after development (5). It occurs by two distinct mechanisms, namely endothelial sprouting and intussusceptive microvascular growth. Endothelial sprouting is based on EC migration into the connective tissues, via degradation of the basement lamina, and formation of a new functional capillary (1, 6, 7), while intussusceptive microvascular growth is the expansion of the existing capillary network, by diving the vessel lumen through the insertion of transcapillary tissue pillars (8, 9).

## Clinical unmet need

Angiogenesis refers to the formation of an adequate, interconnected network of vascular tubes that are a prerequisite to controlled blood flow and is, therefore, a key process in organ growth and development. Indeed, the increased generation of small vessel networks can lead to the regeneration of the tissue environment in ischaemic cardiovascular diseases after injury or atherosclerosis. As the first reports showed decades ago, angiogenesis is also indispensable for tumour growth and transition towards a malignant state capable of metastasis (10). Moreover, recent findings demonstrated that new vessel formation can occur also postnatally, in malignant or ischaemic tissues and in wound healing, and not only during embryonic development, reverting a common assumption accepted for decades (11). Therefore, the inhibition of angiogenesis and vasculogenesis is a promising therapeutic target for cancer, whilst their enhancement holds great potential in wound healing and tissue engineering (12). Recently, anti-angiogenic therapy targeting angiogenic growth factors has been found to have antitumor effect, and some molecules have since been granted approval for clinical use. Targeting of vascular endothelial growth factor (VEGF) and VEGF receptors (using bevacizumab and aflibercept) (13, 14, 15), inhibition of EGF receptor and RAS-ERK pathway (using Tarceva and Cetuximab) (16) and PDGF receptor tyrosine kinases (using sunitinib) (17, 18) are among the most promising therapeutic strategies. Moreover, anti-VEGF and PDGF therapies have been reported to effectively decrease angiogenesis in patients affected by age-related macular degeneration and diabetic retinopathy (14, 19). However, despite the overall preclinical success of targeting VEGF or other angiogenic factors, it is unclear why some patients and several tissue types show resistance or only a limited or heterogeneous response to anti-VEGF compounds (20). The angiogenesis trials in many cases have therefore not reached the significant efficacy anticipated in promising preclinical studies. It suggests that we should refine molecular targets and further understand the underlying complexities of angiogenesis in addition to the mechanisms of action of the agents. To improve patient outcomes and achieve more transformative and effective therapies, we should incorporate new, novel vascular models and validate related predictive biomarkers (e.g. cell cycle, proliferation, energy metabolism and survival) to optimise these therapies specific to different tissues. With this aim in mind, consensus guidelines on angiogenesis bioassays were published in 2018 (21). This is the most exhaustive attempt to provide an in-depth assessment of the approaches used to study angiogenesis and their associated limitations.

## *In vivo* angiogenesis models

To date, vasculogenesis and angiogenesis have largely been studied in mammalian models. The hindlimb ischaemia model, often performed in mice, allows for the reduction in blood flow to the hindlimb to be determined following ligation of the femoral artery. The subsequent enhancement following treatment is often assessed by laser-Doppler perfusion imaging. Although a powerful technique, the endogenous capability of smaller mammals to restore blood flow via post-ischaemic angiogenesis makes an assessment of therapeutic efficacy challenging (22). Zebrafish present themselves as an alternative model to investigate *in vivo* vascular development, in part, due to their relatively low costs resulting in hundreds of fertilised eggs per weeks, capable of fast development. The transparency of the zebrafish embryo allows for real-time visualisation of angiogenesis. Moreover, the zebrafish genome has been sequenced showing a high level of conservation with human coding protein sequences (23). Additionally, this allows for the easy generation of various transgenic lines for the expression of reporter proteins and intravital visualisation with fewer ethical considerations. While zebrafish are broadly applicable to gain a comprehensive understanding of angiogenic processes *in vivo*, however, this model has also some limitations. Fish embryos and larvae are small but constantly grow which poses challenges in longitudinal live imaging. In fact, adult fish lose transparency, dynamics and spatial characteristics of angiogenesis can be therefore technically difficult at this later stage of development. We believe complementary mammalian systems may be necessary for translation of results, given that zebrafish has aquatic, distinct respiratory and cardiovascular architecture. Also, for gene editing approaches, high gene functional conservation between zebrafish and mammals is not complete (24). More recently, lineage tracing approaches have become possible also for mouse models. Mosaic mouse lines with Brainbow (25) or inducible fluorescent genetic mosaic (ifgMosaic) technologies (26) show stochastic expression of multicolour fluorescent proteins specifically in endothelial cells. These models permit fate mapping and 3D visualisation of the clonal dynamics within the vascularised tissue during homeostasis or regeneration as well as in response to angiogenic agents at high cellular and temporal resolution.

## Sources of endothelial cells

During vascular morphogenesis, endothelial cells migrate, differentiate, proliferate and aggregate to form 3D tubular structures, which become new vessels. With all systems, the source of endothelial cells is of fundamental importance to the study results. Primary endothelial cells can form vascular networks *in vitro* and are a common cell source for modelling angiogenesis and study cardiovascular function and diseases, as they can be easily derived and expanded from human circulating blood (endothelial colony-forming cells) (27) or solid tissues, such as cord blood (human umbilical vein endothelial cells, HUVEC) (28), aortic (human aortic endothelial cells, HAEC) (29) and lung tissues (human lung endothelial cells, hLEC) (30). However, scarce availability of tissues, low cell expansion rate and the loss of differentiated phenotype in culture limit their usage (31). Moreover, primary endothelial cells show considerable heterogeneity depending on donor-to-donor variations and tissues of origin, which reflect differences between endothelial populations derived from arteries or veins (20, 32), small or large vessels (33) and normal or tumour vessels. Such heterogenicity must be considered when choosing the cell source for *in vitro* and *in vivo* analyses. Pluripotent stem cells (PSCs), including embryonic (ESC) and induced pluripotent (iPSC), are a promising alternative for overcoming these limitations, as for their ability to self-renew indefinitely in culture and differentiate into different cell fates. In many protocols, first mesoderm specification is induced by the addition of Activin A, and then a vascular specification media is used, supplemented with various combinations of metabolites and growth factors (such as VEGF, FGF-2 and BMP4) which promote the differentiation of hPSC into pluripotent stem cell-derived endothelial cells (hPSC-EC) (34, 35, 36). These cells express endothelial markers, grow as a homogenous cell monolayer with cobblestone morphology, show clonal proliferative potential and can form vessel-like networks *in vitro* and *in vivo* when supported by a hydrogel matrix. Furthermore, arterial phenotype specification was promoted in hPSC-EC cultures exposed to shear stress, as demonstrated by the upregulation of arterial markers Ephrin B2 and Notch1 (37). However, whilst it is now possible to obtain large numbers of hPSC-EC, optimisation of the culture condition is still needed to assess phenotype stability and maintenance of functional properties of hPSC-EC after several passages in culture (30). Finally, mesenchymal stem cells (MSCs) have shown vascular regeneration properties in vitro and in vivo, either by direct differentiation into smooth muscle cells (SMC) and endothelial cells or by the secretion of paracrine factors (38, 39).

## Cell culture media

Given that endothelial cells are highly versatile and are regulated by a multitude of factors, an appropriate selection of growth factors and supplements for angiogenesis assays is critical. To support this effort, various culture media are available, which essentially differ in the composition of supplements. The best characterised angiogenic components used include VEGF, FGF2 (fibroblast growth factor 2), EGF and IGF1 (insulin-like growth factor 1). Alternatively, some media are supplemented with defined concentrations of recombinant growth factors, whereas other media contain bovine-derived endothelial cell growth supplements that are rich in undefined growth-promoting molecules. Media can be also supplemented with hydrocortisone or may contain l-glutamine, heparin and ascorbic acid. However, optimised and standardised media composition for each assay are still warranted (39).

## Phenotypic specification of hESC-EC

Phenotype specification in hPSC-EC cultures is assessed by the expression of common endothelial markers, clonal proliferative potential, monolayer growth and cobblestone-like morphology, together with functional analyses, such as tube formation assay and acetylated low-density lipoprotein (AcLDL) uptake. After purification, cells express CD31, VE-Cadherin, von Willebrand Factor, Neuropilin-1, CD34, VEGFR and laminin alpha 4, as demonstrated by FACS or immunofluorescence analysis (35, 36, 40). Moreover, hPSC-EC showed a similar transcriptional signature and metabolomic profile to primary endothelial cultures (HUVEC, HAEC, and human saphenous vein EC), when gene expression profiles and metabolites were compared by RNA-sequencing and liquid chromatography mass spectrometry respectively (35, 41). However, variations in the expression profiles are expected, depending on the protocol used for the differentiation and time point of the analysis. Importantly, primary EC profiles did not perfectly mimic their native counterpart* in vivo*, raising an important issue about the best control that should be used as comparison for hPSC-EC cultures. Finally, differences in the expression of extracellular matrix components between mature endothelial cells such as HUVEC and hPSC-EC were observed, like differential expression of laminins, perlecan, matrix metallopeptidases MMP1, MMP2 and MMP14, and collagen IV a1 and a2 subunits (36).

## *In vitro* 3D models

In alternative to *in vivo* models, *in vitro* systems can be used to study vasculogenesis and angiogenesis in a controlled, reproducible and cost-effective way. These are easier than animal models and can be used to identify cell types, molecular factors and single steps of vascular morphogenesis. For many decades, 2D-cell culture has been the traditional method of growing cells and studying their interactions. However, investigators have come to understand that the 3D microenvironment determines how cells perceive and interpret biochemical signals, which in turn translates into tissue and organ specificity (42). Recent advances in generating high-fidelity, *in vivo*-like cellular settings can provide us with consistent performance with reduced cell culture artefacts and permit continuous and quantitative imaging. We suggest that scaffold-based cell constructs, as well as organoids, spheroids, hydrogels, and bioprinting (3D) cultures, can facilitate this by providing physiologically relevant models compared to a monolayer (2D) vascular cell culture. Cell behaviour is strongly influenced by cell-cell interactions and microenvironmental cues, i.e. signalling can change when the same cell type is cultured in 3D instead of 2D. 3D cell cultures can be therefore well used in the optimisation of tissue-engineered cell therapy manufacturing, including adequate vascularisation. To study the effect of biomaterials and growth factors that direct the process of angiogenesis, numerous models have been established (43, 44) (Table 1). 3D cell systems have been proved to be advantageous compared to 2D as they allow superior reproduction of the physiological environment of cells *in vivo*, by maintaining cell-to-cell and cell-to-matrix interactions that control development, differentiation and signalling (45). 3D cell culture methods can be divided into two classes: scaffold-based and scaffold-free models.

**Table 1:** *In vitro*, *ex vivo* and *in vivo* 3D models/techniques to study angiogenesis.

Assay	Application	Considerations	References
*In vitro* assays			
Cell culture wound closure assay	Assessing EC migration		(71, 72)
Boyden chamber	Assessing EC migration		(73)
Collagen lumen assay	Assess tube and lumen formation	Limited useful time frame (72 h), due to contraction of gels	(74)
Fibrin bead assay	Assessing sprouting and lumen formation		(75)
Vascularised micro-organ platform (VMO)	Assessing all steps of vascular formation. In VMO, endothelial cells form a luminised, perfusable, vascular network that respond to shear stress		(76)
EC-coculture spheroids	Assessing the interplay between endothelial cells and mural cells and special growth of vascular structures, in physiological and pathological conditions. Drug screening		(58, 77, 78)
*Ex vivo* (organotypic assays)			
Aortic ring assay	Assessing the outgrowth of endothelial cells (and other cells) from aortic explants. This system allows to study sprouting from a native tissue, and to test the efficacy of pro- and anti-angiogenic molecules in a physiological environment	Lack of flow. High results variability depending on background and age of the animal or donor	(79, 80, 81)
Retinal explant assay	Studying microvessel formation and its remodelling		(82, 83)
*In vivo*			
In-ovo chorioallantoic membrane assay	Assessing the vascularisation of allografts or xenografts. Testing the effect of pro-angiogenic and anti-angiogenic treatments	The chicken embryos hatch around developmental day 21 and experiments cannot exceed day 18. Limited quantification to evaluate the results	(84)
Corneal angiogenesis assay	Studying molecular and cellular mechanisms of angiogenesis. Testing the effect of pro-angiogenic and anti-angiogenic treatments	It requires considerable technical skills	(85, 86)
Matrigel plug assay	Assessing the effect on vascularisation of cells and substances mixed with Matrigel and injected subcutaneously		(87)

## Scaffold-based models

Hydrogels recapitulate the molecular environments of ECM *in vivo,* giving cells a 3D support matrix that they can interact with and are capable of remodelling. Hydrogels are often used *in vitro* to create models of angiogenesis, and they have shown potential for the study of both vascular morphogenesis and the preclinical testing of drugs (46). Different types of synthetic hydrogels have been shown to support angiogenesis, both of natural (alginate, hyaluronic acid, fibrin and gelatin) and non-natural origin (polyethylene glycol and poly (lactic-co-glycolic acid)). Collagen I and Matrigel are commonly used natural ECM-based hydrogels, although their animal origin, batch-to-batch variation and unknown composition limit their application. The advantage of hydrogels is their ability to encapsulate and release bioactive agents. Immobilisation of regulatory factors, such as RGD peptides and VEGF, in hydrogels, promote vascular differentiation of encapsulated cells (47). More recently, combining hydrogels, bioactive agents and a co-culture system have provided a model of tumour angiogenesis to further understand the role of endothelial cells in the tumour microenvironment (48). Hydrogel can be also incorporated into microfluidic systems (49). However, the composition and the density of fibres in the hydrogels have severe effects on the assembly of the microvasculature (50). For example, while increasing the fibre density usually improves the integrity of the hydrogels, however, augmenting collagen density by one order of magnitude decreased EC sprouting distance by 50% (50, 51). At the same time, the concentration cannot be too low, as low-density hydrogels do not provide enough support for EC migration (52). Moreover, matrix biodegradability was shown to profoundly alter EC migration speed and efficiency, with less-biodegradable hydrogels encouraging EC sprouting compared to more-degradable ones (53, 54). Finally, stiffness of the substrate can also influence EC proliferation, signalling and differentiation (55), Therefore, ECM protein composition and concentration must be carefully optimised to ensure that hydrogels promote vascularisation while maintaining their structural integrity.

## Scaffold-free models

However, the advent of spheroids systems has provided a new method to study angiogenesis *in vitro*. Here, the addition of synthetic-derived scaffold to mimic the ECM is not required, and instead, cells are grown as self-assembling aggregates. In these 3D systems, the cells grow, differentiate and deposit their own ECM, in a way that closely recapitulates the *in vivo* physiological conditions. Spheroids can be used to study the physiological spatial growth of vessel structures, the cell-cell interactions, and as a platform for drug development and discovery (30). Hybrid spheroids, generated by co-culturing two or three types of cell, are useful models to study the interaction between endothelial and tumour/stroma cells in different *in vitro* models of cancer (56, 57) or between endothelial and mural cells in angiogenesis/vasculogenesis. Analysis of endothelial cell/MSC spheroids showed that MSC participated in the formation and stabilisation of luminal tubular structures, similarly to pericyte-like cells (58, 59). Many methods can be used to generate the spheroids including hanging drops, low adhesion plates and self-organising 3D vessel-like structures (vascular organoids) (60). Notwithstanding the versatility and cost-effectiveness of scaffold-free models, the cell density and dimensions of the spheroids must be carefully monitored, as oxygen and nutrients diffusion to the core might be affected. Consequently, time in culture may be as well limited (14). Finally, as 3D culture systems become more popular and continue to evolve, more sophisticated imaging techniques need to be developed to image and analyse thick samples (61).

## Microfluidic devices for *in vitro* 3D assays

A major challenge remains to find a 3D system capable of mimicking the physiological and pathological conditions of the *in vivo* systems. We know that *in vivo* the vascular endothelium is continuously exposed to shear stress and hypoxia, and EC physiology varies in response to different flow patterns and rates compared to static culture conditions (62). Moreover, for 3D culture systems, it is essential to improve medium diffusion, oxygen and nutrients supply to ensure cell survival in the core of the microtissues. Application of flow to angiogenic culture models is the most distinctive advantage of using microfluidics, other than reducing costs and complexity of the experiments and minimizing the volume of reagents (63, 64). Furthermore, *in vitro* microfluidic systems allowed to study in a controlled manner the effects of normal and disturbed flow on endothelial cells and the interactions between endothelial cells, supporting cells (pericytes and SMC) and platelet (65, 66, 67, 68).

However, the designing of microfluidic devices requires specific competencies which are usually beyond the expertise of the final operators, and some limitations remain in the application of microfluidic systems (69, 70).

## Conclusions

Recent progress in 3D models of vascularisation has allowed for invaluable advancements in our knowledge of angiogenesis/vasculogenesis. In this review, for each model we reported advantages and limitations. Different tests should be used together to obtain the maximum of information. *In vitro* tests, although informative, are yet unable to divulge the more complex interactions between endothelial cells and other cellular constituents of the microvessel wall or the response to flow or shear stress which plays an important role in vascular mechanobiology and should be complemented with *in vivo* models.

## Declaration of interest

The authors declare that there is no conflict of interest that could be perceived as prejudicing the impartiality of this review.

## Funding

This work was supported by the BHF Regenerative Medicine Centre, the Medical Research Councilhttp://dx.doi.org/10.13039/501100000265 (MR/R025002/1) and the Hungarian National Research, Development and Innovation Fund (NVKP-16-1-2016-0017 and 128444).
